# Preoperative anemia and total hospitalization time are the independent factors of preoperative deep venous thromboembolism in Chinese elderly undergoing hip surgery

**DOI:** 10.1186/s12871-020-00983-2

**Published:** 2020-04-02

**Authors:** Long Feng, Longhe Xu, Weixiu Yuan, Zhipeng Xu, Zeguo Feng, Hong Zhang

**Affiliations:** 1 Medicine School of Chinese PLA, No.28, Fuxing Road, Beijing, 100853 China; 2 Department of Anesthesia, Hainan Hospital of Chinese PLA General Hospital, No.80, Jianglin Road, Sanya, 572000 China; 3 Department of Anesthesia Operation Center, Chinese PLA General Hospital, No.28, Fuxing Road, Beijing, 100853 China

**Keywords:** Anesthesia, Deep vein thrombosis, Hip fracture, Hospitalization time, Anemia

## Abstract

**Background:**

This study was designed to explore the prevalence and risk factors of preoperative deep venous thromboembolism (DVT) in Chinese elderly with hip fracture.

**Methods:**

From January 1, 2012, to December 31, 2018, 273 elderly patients over 70 years old with elective hip surgery were collected from the electronic medical records. Collected data included demographic characteristics, comorbidities, ASA classification, types of previous operations, types of anesthesia, operation time, fracture to operation time, preoperative hemoglobin level, anemia, blood-gas analysis, cardiac function, whether transfusion, preoperative hospitalization, postoperative hospitalization, electrocardiograph, lower limb venous ultrasonography and total hospitalization time.

**Results:**

In these 273 patients, 15(5.6%) had ultrasonography evidence of DVT in affected limbs before surgery. Three of all patients received an temporary inferior vena cave filter placement preoperatively. Fracture to surgery time, preoperative hemoglobin level, anemia, preoperative hospitalization, pulmonary disease and total hospitalization time were statistically different between DVT group and non-DVT group (*P* < 0.05 for all). Moreover, preoperative anemia (OR: 0.144, 95%CI: 0.026–0.799, *P* = 0.027) and total hospitalization time (OR: 1.135; 95%CI: 1.023–1.259, *P* = 0.017) were the two independent risk factors for preoperative DVT.

**Conclusion:**

Preoperative anemia and total hospitalization time were independent risk factors for venous DVT in Chinese elderly with hip fracture.

## Background

Venous thromboembolism (VTE) including deep vein thrombosis (DVT) and pulmonary embolism (PE) is a serious and preventable complication after hip fracture [1–4]. The risk for VTE among patients undergoing major orthopedic surgery, particularly hip fracture surgery, is the highest among all surgical patients. It has been reported that preoperative DVT had an incidence of 6–9% in patients with hip fracture receiving surgery within the 48 h, whereas the rate could be raised to 54.5–62% when there was a delay for more than 48 h [5]. Pedersen et al. [6] have proposed that hip fracture was associated with increased subsequent risk of VTE in a population-based cohort study of 110,563 patients with incident hip fracture. The risk of VTE increased 17-fold in the first 30 days after hip fracture, declining to a 2.1 fold increase from 31 to 365 days following hip fracture. Risk factors for VTE include age, obesity, chronic obstructive pulmonary disease (COPD), atrial fibrillation, anemia, depression, trauma, total knee arthroplasty, hypercoagulable states and postoperative complications [7]. Shahi et al. [8] have also pointed out that the advanced age (greater than 70 years old, OR: 1.3, 95% CI:1.1–1.4) is the risk factors for developing in-hospital VTE. However, limited studies has been performed to observe the risk factors of preoperative DVT in Chinese elderly over 70 years old with hip fracture. Thus, the purpose of this study was to explore the prevalence and risk factors of preoperative DVT in Chinese elderly over 70 years old with hip fracture.

## Methods

**Fig. 1:**
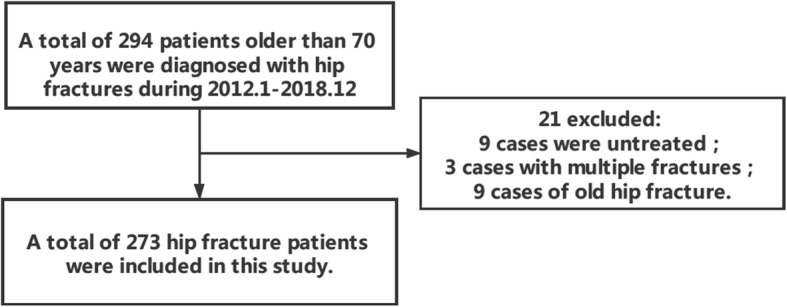
Exclusion criteria and the number of studies were included in this study

This retrospective single-center study included 273 consecutive patients over 70 years old with hip fracture and elective surgery in Hainan Hospital of Chinese People’s Liberation Army General Hospital from January 1, 2012 to December 31, 2018. Exclusion criteria for this study included age < 70 years, multi-type of fracture and conservation treatment (Fig. 1). All data were collected from the electronic medical records. Collected data included demographic characteristics, comorbidities (including diabetes, hypertension, stroke, ischemic heart disease, arrhythmia, congestive heart failure, and COPD), hemoglobin level, erythrocyte sedimentation (ESR), D-dimer, ASA classification, types of surgeries, types of anesthesia, preoperative hospitalization, postoperative hospitalization, whether transfusion, operation time, fracture to operation time, preoperative hemoglobin level, anemia (the anemia was defined as hemoglobin below 120 g / dL in male and 110 g / dL in female), blood-gas analysis, cardiac function, electrocardiograph, preoperative lower limb venous ultrasonography and hospitalization time. Types of hip fractures included the femoral neck, intertrochanteric, subtrochanteric, and proximal shaft fractures.

Performed surgeries included hip replacement and fixation procedures. All patients with hip fractures were routinely treated with a low molecular weight heparin sodium daily to prevent DVT after hospitalization. In addition, all patients underwent routine ultrasound examination of the lower extremities before surgery and before discharge. DVTs were classified into three types: central type, peripheral type, and mixed type. Central type referred to thrombus occurring proximal to the knee in the iliacs, superficial femoral and/or femoral veins. Peripheral type was defined as thrombosis distal to the knee in the posterior tibial veins or peroneal veins. DVT was classified as mixed type when involving the whole deep venous system of lower limb. Ultrasonography of lower limb veins was usually performed again before leave hospital. The diagnosis of DVT was according to Robinov criterion, which are included the following four parts: 1. In constant filling defects, thrombi are constant in appearance, and tend to be sharply delineated; 2. Abrupt termination of the opaque column occurs at a constant site in a vain, either above or below the obstruction; 3. Nonfilling of the entire deep system or portions thereof when proper technique is used is abnormal and usually due to phlebitis; 4. Diversion of flow, representing collateral flow, is the counterpart if the nonfilling described above [9]. Besides, the anemia in this study was defined as hemoglobin below 120 g / dL in male and 110 g / dL in female.

### Statistically analysis

Continuous data were presented as the means and standard deviations (SD). Categorical data were presented as the numbers and percentages. By comparing the DVT group with the non-DVT group, when performing univariate logistic regression analysis when *P* values < 0.05 is a risk factor. When the factors *P* values is < 0.1, a multivariate analysis is performed. These risk factors were then included in multivariate logistic regression analyses to detect the risk factors independently affecting the DVT. Odds ratios were displayed with a 95% confidence interval if the *p* < 0.05. *P* < 0.05 was considered statistically significant. All data were analyzed in Statistic Package for Social Science (SPSS) version 19.0 (SPSS Inc., Chicago, USA).

## Results

### Clinical characteristics

All patients had an average age of 78 ± 11 years, and 57% were women. Among the 273 patients, 1 underwent surgery within 24 h, 16 within 48 h, 57 within 72 h and the 199 more than 72 h. The mean time to surgery was 99.6 ± 22.1 h. There were 3.4% of patients with surgery within the 48 h after the fracture. Fifteen patients (5.6%) had limb DVT (10 cases were peripheral type, and 5 cases were central type). Two of the DVT (1 peripheral type and 1 central type) occurred 48 h before surgery, and the other 13 occurred after 48 h from the time of hip fracture. No PE occurred in the perioperative period.

### Univariate analyses

In the univariate analysis, fracture to surgery time, preoperative hemoglobin level, anemia, pulmonary disease, preoperative hospitalization and total hospitalization time were statistically different between DVT group and non-DVT group (*P* < 0.05 for all). There were no statistical difference between two groups in age, sex, diabetes, hypertension, stroke, ischemic heart disease, arrhythmia,whether transfusion, congestive heart failure, postoperative hospitalization, international normalized ratio (INR) and ESR (*P* > 0.05) (Table 1).

**Table 1:** Factors associated with the development of perioperative DVT

Variables	DVT group	Non- DVT group	***P*** value
Age(mean years±SD)	78 ± 4	78 ± 5	0.911
Male sex (%)	2	70	0.330
BMI(mean ± SD)	22.9 ± 4.1	23.1 ± 4	0.910
EF(%)	60.5 ± 1.1	60.6 ± 3.7	0.661
**Comorbidities**			
Hypertension	5	117	0.303
Coronary artery disease	1	42	0.478
Diabetes	3	58	0.529
Arrhythmia	2	32	0.600
Cerebral infarction	2	32	0.583
Pulmonary disease	3	15	0.074
Anemia	13	133	0.014
PH(mean ± SD)	7.43 ± 0.02	7.49 ± 1.22	0.861
Hb(mean ± SD)	107.1 ± 10.1	115.6 ± 18.7	0.007
PCO_2_(mean ± SD)	37.7 ± 4.4	39.8 ± 5.7	0.212
PO_2_(mean ± SD)	76.2 ± 9.6	75.9 ± 17.9	0.490
D-dimer(mean ± SD)	4860 ± 7824	3373 ± 7380	0.095
ESR(mean ± SD)	42.0 ± 18.6	33.9 ± 19.7	0.113
**ASA classification**			
I-II	6	106	0.814
III-IV	9	143	0.814
**Anesthesia method**			
General anesthesia	2	46	0.859
Epidural anesthesia	0	16	0.609
Regional nerve block	10	151	0.892
General + nerve block	3	33	0.443
Injure to operation time(day)	18.1 ± 12.1	13.7 ± 36.3	0.002
**Type of operation**			
Hip replacement	1	25	1.000
Femoral head replacement	4	93	0.386
Bone nail	10	128	0.270
Operation time(min)	103.1 ± 53.4	99.7 ± 47.4	0.901
Preoperative hospitalization(day)	8.1 ± 3.2	6.4 ± 3.2	0.039
Postoperative hospitalization(day)	12.4 ± 4.5	10.0 ± 6.4	0.159
In-hospital time(day)	20.5 ± 4.5	16.5 ± 7.3	0.001
Blood loss(ml)	365 ± 464	258 ± 220	0.898
Whether transfusion	2	36	0.904

*BMI* Body mass index, *ASA* American society of anesthesiology, *ESR* Erythrocyte sedimentation rate, *EF* Ejection fraction

### Multivariate analyses

Multivariate logistic regression analyses confirmed that preoperative anemia (OR: 0.144, 95% CI: 0.026–0.799, *P* = 0.027) and total hospitalization time (OR: 1.135; 95%CI: 1.023–1.259, *P* = 0.017) were the two independent risk factors for preoperative DVT (Table 2).

**Table 2:** Multivariate logistic regression analysis

Risk factors	OR	95%CI	***P*** value
Hemoglobin level	0.965	0.93–1.001	0.056
The length of stay	1.135	1.023–1.259	0.017
Pulmonary disease	1.135	0.117–10.973	0.913
D-dimer	1.000	1.000–1.000	0.081
Injure to surgery time	1.005	0.984–1.026	0.670
Anemia	0.144	0.026–0.799	0.027
Preoperative hospitalization	1.129	0.987–1.292	0.076

*OR* Odds ratio, *CI* Confidence interval

*P < 0.05 was considered statistically significant

## Discussion

This study demonstrated that the overall incidence of DVT after hip fracture was 5.6%, and no PE occurred in all patients. In addition, multivariate logistic regression analyses indicated that preoperative anemia and total hospitalization time were the independent risk factors for preoperative DVT after hip fracture.

Hip fracture is one of the most common orthopedic conditions. The risk of VTE in patients with hip fracture is substantial, which is the second most frequent complication of surgery. Reboerts et al. [3] and Hefley et al. [4] have reported that the incidence of DVT was about 6–9% in patients with hip fracture. In addition, Wong et al. [10] have been reported that the incidence of VTE was 6.4% after proximal hip fracture in Singapore. Mok et al. [11] have also reported that the incidence of VTE was 8% after proximal hip fracture in Hong Kong. All above results are the same to our results. Furthermore, delayed surgery for these kinds of patients is known to be one of the most important factors contributing to the high incidence of preoperative DVT [4]. Hip fracture surgery should be performed within 48 h after fracture [12]. However, in clinical work, targeting within the 48 h, even in the 24 h, represents a significant change in practice because 66% of the patients did not receive surgery within time frame [13]. Only 3.4% of patients in our study completed surgery within 48 h after fracture, because the multi-disciplinary consultation and preoperative evaluation are often required owing to the prevalence of severe comorbidities in these patients but assessing appropriately.

The incidence of anemia at admission in individuals with hip fracture is high, varying from 12.3% with hemoglobin level less than 10 g/dL to 40.4% with hemoglobin level less than 12 g/dL [14]. Anemia is associated with increased mortality, increase VTE risk, prolong admission, higher readmission rate and increased mortality rate in patients with hip fracture [15–20]. Furthermore, most patients in this study often had cardiovascular disease (28%) before surgery, which reminded that we should actively correct a severely decreased preoperative hemoglobin of less than 9 during perioperative low hemoglobin in order to reduce the risk of cardiovascular events. Because the most frequent cause of death after hip fracture surgery is cardiovascular diseases [21]. The lower hemoglobin level at admission is not owing to bleeding from trochanteric fracture, but reflects the anemia before the injury. It is known that the anemia and low hemoglobin concentrations were significantly associated with frailty [22]. Frailty has been shown to predict adverse outcomes in older surgical patients, which is related with more postoperative complications, length of stay, and greater morbidity and mortality [22–25] . However, frailty is a common status among hip fracture patients and seriously affect quality of life on these patients [23, 24]. Chen et al. [25] study found that the frailty state of elderly patients with hip fracture surgery can significantly increase major adverse events, including mortality, readmission, and postoperative emergency room visits. Inoue et al. [26] also pointed that the frailty can be assessed as a predict short-term functional recovery during the acute phase in patients with hip fracture. Therefore, early identification of prefracture frailty in patients with a hip fracture is important for prognostic counselling, care planning and the tailoring of treatment [27].

The total estimated number of hip fracture in Asian countries will increase from 1.12 million in 2018 to 2.56 million in 2050 [28]. Hip fractures are related to increased morbidity and adverse clinical outcomes during hospitalization and discharge are common and costly occurrences [29]. It is logical to perform surgery as early as possible (Best within 48 h after hip fracture) in order to avoid these complications, especially to reduce the risk of VTE. Optimal strategies for thromboprophylaxis after hip fracture also include use one of the following antithrombotic prophylaxis (Low molecular weight heparin, Fondaparinux, Low dose unfractionated heparin, et al) for a minimus of 10 and/or 14 days, or an intermittent pneumatic compression device [30]. In addition to above measures, more and more evidence have suggested that comprehensive geriatric assessment decreased the risks of complications after hip fracture [31],which is not delaying surgery but assessing appropriately. Kammerlander et al. [32] have been pointed out that the interdisciplinary team could achieve the lowest in-hospital mortality rate (1.14%), the lowest hospitalization time (7.39 days) and the lowest mean time to surgery (1.43 days). Besides, comanaged geriatric fracture center program that has resulted in lower than the predicted hospitalization time and readmission rates, with short time to surgery, low complication rates and low mortality [33, 34]. A previous study has also pointed out that the mean postoperative length of stay was 5 days in the USA and 34 days in the Japan, and the risk of death after hospital discharge was doubled in the USA in comparison with Japan [35]. Because shorter length of stay after hip fracture is associated with increased risk of death after hospital discharge, but only among patients with length of stay of 10 days or less [36]. Therefore, it is prudent to prolonged hospital stay for patients at high risks after hip fracture surgery. Furthermore, European and North American studies have also shown that care provision by more nurses with at least bachelor’s degrees are associated with lower mortality after surgery [37, 38]. Physical therapy also important to enhance functional capacity and postpone the need for institutional care, and diminish the use of social and health care services for the older with signs of frailty or with a recent hip fracture [39].

### Limitations

Our study has some limitations. First, this study was a single-center retrospective analysis and all data were retrospectively collected. A multi-center randomized controlled trial is needed in the future. Second, only 5.6% of patients in this study were found to have DVT before surgery, some maybe were missed on ultrasonography. Third, our study not evaluate the postoperation and long term morbidity, such as the arrhythmia, myocardial infarction and pneumonia. Four, this study not mention the importance of physical therapy for reduce the incidence and severity of frailty and mortality.

## Conclusion

In order to reduce the risk of DVT, it is currently agreed that elderly hip fracture patients should be operated as soon as possible, preferably within 48 h after the fracture. However, for critically ill patients, comprehensive geriatric assessment is not about delaying surgery but assessing appropriately. Besides, cardiovascular diseases are often associated with such patients before operation. Active correction of severe anemia of < 9 and frailty is also beneficial to reduce the risk of cardiovascular events, morbidity and mortality during perioperative period. In addition, prevention and minimize the risk of DVT after postoperatively should be mobilization with active physical therapy, chemical prophylaxis against VTE (such as Low molecular weight heparin, et al) for a minimus of 10 to 14 days, and surveillance with screening ultrasonographies. For high-risk patients, the length of hospital stay should be appropriately extended, and aggressive postoperative medical care and physical therapy also should be received.

## Data Availability

The datasets used and/or analysed during the current study are not publicity available. All data are available from the corresponding author upon reasonable request.

## Abbreviations

DVT: Deep vein thrombosis
VTE: Venous thromboembolism
ASA: American Society of Anesthesiologists
PE: Pulmonary embolism
ESR: Erythrocyte sedimentation
BMI: Body mass index
Hb: Hemoglobin
EF: Ejection fraction
